# Comprehensive immunoprofiling and systematic adjuvant comparisons for identifying suitable vaccine: Adjuvant pairings

**DOI:** 10.1080/21645515.2023.2223503

**Published:** 2023-06-21

**Authors:** Nancy Vázquez-Maldonado, Halonna R. Kelly, Wolfgang W. Leitner

**Affiliations:** Division of Allergy, Immunology and Transplantation, National Institute of Allergy and Infectious Diseases, National Institutes of Health, Bethesda, MD, USA

**Keywords:** Adjuvant, immunostimulators, reactogenicity, immunoprofiling

## Abstract

Adjuvants are critical components of vaccines that enhance the host immune response to the vaccine antigen, however, only a small number of adjuvants are used in vaccines approved for human use. This is in part due to the slow process of novel adjuvants advancing from preclinical models to human studies, and modest mechanistic insights obtained using standard immunological methods to justify selection of a particular adjuvant for clinical evaluation. Here, we discuss several aspects of current adjuvant research and strategies to better assess the complex pathways triggered by adjuvant candidates that can increase adjuvanticity and vaccine efficacy while minimizing reactogenicity. We propose a more systematic use of broad immunoprofiling, coupled with data integration using computational and mathematical modeling. This comprehensive evaluation of the host immune response will facilitate the selection of the most appropriate adjuvant for a vaccine, ultimately leading to the expeditious evaluation of novel adjuvants for vaccines against emerging infectious diseases, which will prove especially valuable during a pandemic where speed is of the essence when developing vaccines.

## Introduction

Exogenous immunostimulators are used as adjuvants for modern vaccines, however, in the United States, the number of adjuvants in vaccines licensed for human use is still surprisingly small. Countless novel adjuvants are in preclinical development, and many novel adjuvants are evaluated in early-stage clinical trials, yet adjuvant selection for a particular vaccine continues to be mostly an empirical process. “Rational adjuvant selection,” the targeted pairing of an adjuvant with a vaccine antigen, is an attractive, but still elusive concept. Selection of the most appropriate adjuvant can determine the efficacy of a vaccine candidate, based on both strength and profile of the immune response being triggered. Even though data on the preclinical and clinical performance of different adjuvants with various antigens are noted in the literature, for example dmLT,^a^ GLA,^b^ or Advax,^c^ it is difficult to compare immune characteristics mediated by the different adjuvant candidates when the underlying studies differ regarding the antigens used, immunization regimens (i.e., dose, route of immunization), vaccine formulation, animal model, and immunological and clinical readouts. Systems vaccinology studies that include the analysis of a wide range of immune readouts, such as the assessment of innate and adaptive immune signals, phenotypic and spatial analyses of immune and nonimmune cell populations, and various other factors, prove more valuable at predicting vaccine efficacy than analyses that include only one or few parameters.^1,2^ This type of comprehensive analysis that pinpoints the contribution of the adjuvant to the observed immune profile versus the impact of the vaccine antigen alone, in different model systems, utilizing a broad range of immune readouts will result in highly complex data sets that require computational data integration to establish an adjuvant’s “immune fingerprint.” Cataloging the immune fingerprints of different adjuvants would provide immunogenicity and reactogenicity profiles in a standardized fashion.

Here, we discuss current limitations of key aspects and stages of adjuvant research: adjuvant discovery, preclinical evaluation, reactogenicity, and species-specific differences in the immune response. We offer suggestions on how to incorporate immunoprofiling into these aspects; and highlight how the establishment of comprehensive immune profiles induced by different adjuvants could expedite and standardize vaccine adjuvant.

## Adjuvant immunoprofiling: Adjuvant discovery

Modern adjuvant discovery involves systematic *in vitro* screening of compounds, with one (or few) marker(s) as the readout to determine whether a new compound has the potential to be used as an adjuvant.^3–5^ This approach assumes that expression of a specific signaling molecule (e.g., NF-_ƙ_B activation) or cytokine (e.g., IL−6 or TNFα) correlates with adjuvanticity *in vivo*. However, since a single *in vitro* parameter is unable to reliably predict *in vivo* adjuvanticity, the approach can result in a significant number of false positive hits, and potentially many false negatives. The high failure rate is due to a lack of defined *in vitro* correlates of *in vivo* adjuvanticity. Multiplex assay platforms that expand the number of cytokines being assessed partially address the issue,^6^ but this approach still relies on a limited number of parameters that may not necessarily correlate with *in vivo* adjuvanticity and can be prohibitively expensive when scaling up an adjuvant screening campaign.

**Figure 1.**
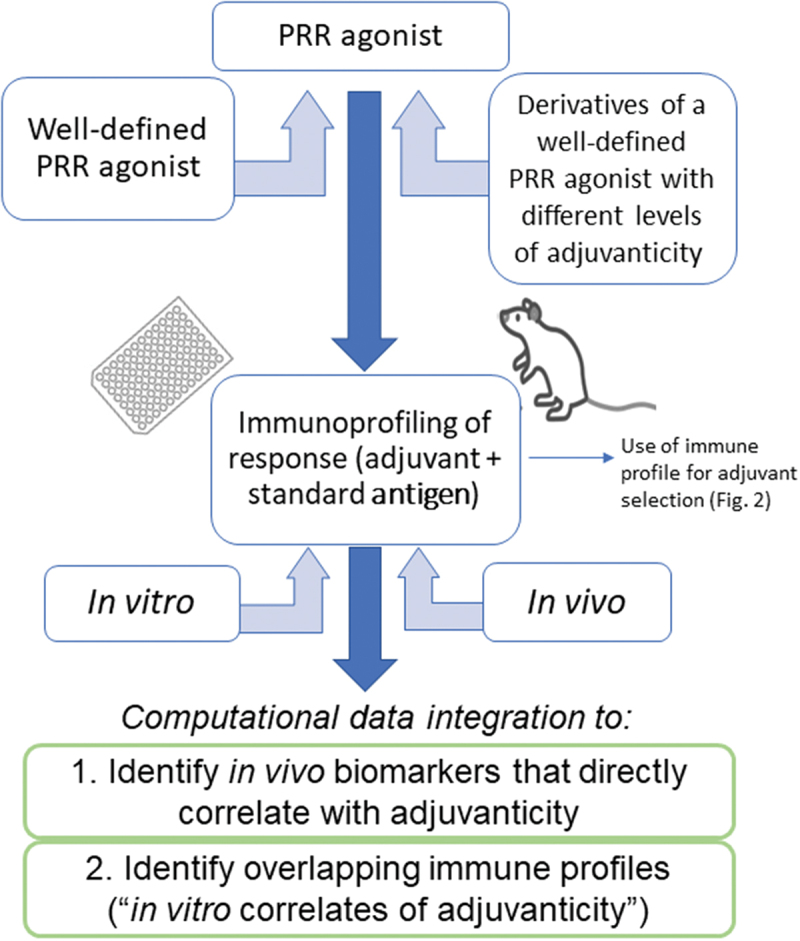
Proposed approach for establishing *in* *vitro* correlates of adjuvanticity to aid in adjuvant discovery. Example shown is for a generic PRR agonists and involves the use of well-established adjuvants targeting the PRR as well as derivatives of such compounds to establish a range of response patterns *in* *vivo* as well as *in* *vitro*. Computational data integration is used to a) identify biomarkers that directly correlate with adjuvanticity *in* *vitro* and b) help identify corresponding, minimal response patterns *in* *vitro*. Applying this approach to different PRR agonists may also identify an *in* *vitro* adjuvanticity profile that applies to a wide variety of types of adjuvants (“pan-PRR adjuvanticity profile”). Comparing the profile induced by the adjuvant alone and different adjuvant/antigen formulations provides further insights into the contribution of the antigen to the innate immune response profile. Once established, immune profiles of adjuvants can inform rational adjuvant/antigen pairing (see Figure 2).

We propose that adjuvant researchers employ a multi-step adjuvant discovery process (Figure 1), that couples *in vivo* immunoprofiling studies with different adjuvants that have well-documented *in vivo* adjuvanticity, paired with “standard” antigens, to identify *in vivo* markers (not limited to cytokines and chemokines) associated with adjuvanticity. The inclusion of antigens in the profiling study assures that contributions of the antigen to the overall immune profile are recognized. To facilitate and refine the process of identifying *in vitro* immune markers that directly correlate with *in vivo* adjuvanticity, derivatives of strong adjuvants could be used, such as those that are generated in the process of structure-activity-relationship studies of novel adjuvants.^7,8^ The derivatives may have different levels of adjuvanticity and potentially mediate different immune profiles from which common biomarkers that directly correlate with adjuvanticity may be deduced. Computational modeling can help identify the minimally required biomarker signature suitable for predicting *in vivo* adjuvanticity (i.e., a candidate adjuvant’s “*in vivo* adjuvanticity profile”) and be employed as an *in vitro* tool for identifying novel adjuvants. Considering the complexity of innate immune receptors, signals, and downstream effector molecules, it is likely that different (but overlapping) patterns will emerge from such analyses depending on the pattern recognition receptor (PRR) being targeted. For example, immune signatures shared between agonists of different PRRs would identify a pan-PRR adjuvanticity profile.

**Figure 2.**
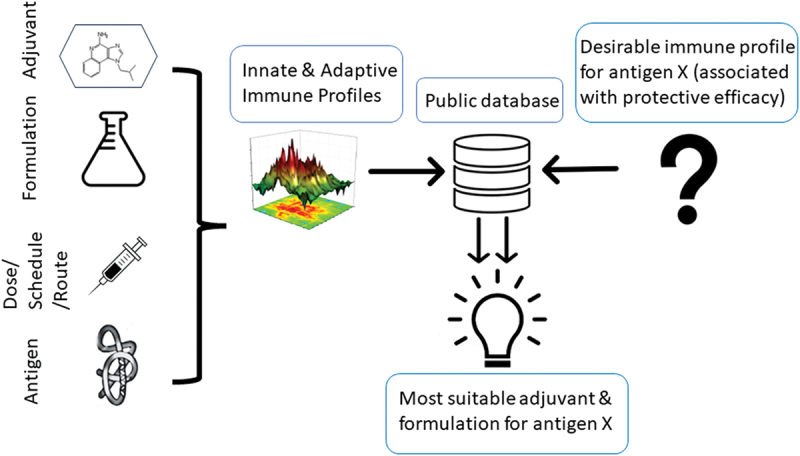
Proposed establishment of publicly accessible data repositories for immune profiles of adjuvants to inform rational adjuvant selection. An adjuvant’s innate and adaptive immune profile is affected not only by the adjuvant itself, but also formulation (delivery vehicle), the immunization (dose, schedule, and route) and the co-delivered antigen. Varying these parameters helps identify the aspects of the profile that are imprinted by the adjuvant itself. Knowing the desirable immune profile that a vaccine (that is based on “antigen X”) should induce, the database will facilitate the identification of the most suitable adjuvant and formulation in silico, thus bypassing the need to conduct laborious vaccination studies with multiple adjuvants.

The application of immunoprofiling extends beyond providing insights into an adjuvant’s mechanism of immune enhancement and understanding differences between different classes of immunostimulators. The ultimate goal in vaccine research is the rational rather than empirical pairing of antigen and adjuvant by selecting adjuvants and formulations capable of driving the immune profile associated with protective efficacy (Figure 2). While immune correlates of protection are still poorly understood for most diseases and subject of ongoing research, they are also not necessarily static (e.g., depend on the antigen). Nevertheless, certain desirable features of vaccine-induced immune responses are known for a variety of pathogens (e.g., need for extensive somatic hypermutations in anti-HIV gp120 antibodies). Building databases to house the immune profiles of adjuvants will assure that the information for rational adjuvant selection is available as more insights into immune correlates of protection are generated.

## Adjuvant immunoprofiling: Preclinical evaluation

Antibody titers currently serve as the primary marker and predictor of a promising vaccine-induced response in preclinical models of adjuvant development. The choice of this readout is not only driven by the fact that antibody titers are the primary accepted correlate of protection for most licensed vaccines (reviewed in ref.^9–11^ for influenza, hepatitis B, and malaria respectively), but currently, it also represents the most technically feasible readout. For some vaccines, a functional activity of antibodies has been described as a correlate of protection, e.g., such as opsonophagocytic activity in case of malaria vaccines.^12^ Relying on antibody titers as the main parameter for choosing a vaccine adjuvant is limiting because it does not consider other immune parameters that may contribute to vaccine efficacy,^13^ nor does it help distinguish adjuvants from each other. More useful parameters include a broader assessment of the antibody repertoire, such as antibody avidity and breadth of epitope specificity, and the analysis of vaccine-induced T cells, particularly CD8 T cells. The mere assessment of antibody titers may also be misleading since antibody responses even against immunodominant epitopes may not correlate with efficacy, such as in the decoy antigens/epitopes of the HCV envelope glycoprotein.^14^ In addition, while studies to identify *in vivo* markers of adjuvanticity have been conducted in humans, for example with adjuvants from the GSK Adjuvant System (AS-series),^15^ such studies are complicated and expensive, which highlights the need for preclinical models.

Therefore, we are advocating for comprehensive immunoprofiling in preclinical studies to determine the suitability of antigen/adjuvant combinations to inform decisions to move forward to clinical development. Computational integration of these data, together with results from efficacy studies can help identify correlates of protection that can be used in further studies of the vaccine to refine readout methods in a targeted manner. While animal models, especially small animals, should not be used to narrow the choice to a single adjuvant for a clinical trial of a vaccine due to species-specific differences in the immune response (discussed below), profiling data from preclinical adjuvant comparison studies can still help in down-selecting a small number of adjuvant-antigen combinations to be tested in humans. Human organoid systems are still early in development and not commonly used but could be a valuable non-animal tool for down-selecting adjuvants in the future.

## Adjuvant immunoprofiling: Reactogenicity

Reactogenicity is defined as the inflammatory response induced by a vaccine. Symptoms of reactogenicity can vary, ranging from localized injection-site pain, redness, and swelling, to systemic symptoms, such as headache, fever, or myalgia. The primary concern that slows advancement of novel adjuvant candidates into clinical trials is the risk of reactogenicity and adverse events, mostly as a result of the induction of innate immune cells, triggering inflammatory pathways. Traditionally, strong inflammation is associated with good immunogenicity, although there is little evidence to support this association.^16^ Reactogenicity can be mitigated through 1) adjuvant design and modifications that skew the response toward signals that contribute to adjuvanticity (e.g., the “detoxification” of LPS resulting in derivatives such as MPL;^17^ 2) improved formulation, such as the targeting of immunostimulators to secondary lymphatic organs, which prevents systemic and injection site availability (e.g., through chemisorption to a carrier, such as Alum, in the case of Alhydroxiquim-II^d^); or more recently, 3) the use of selective inhibitors of inflammatory signals as co-adjuvants.^18^ Despite these mitigating steps, time and resources often are wasted on novel immunostimulatory compounds that turn out to have unacceptable safety profiles in preclinical models. Furthermore, the ability of animal models to predict adjuvant:vaccine safety concerns in humans is highly limited. For example, for Shingrix or mRNA-based COVID vaccines,^19^ conventional mouse models do not replicate or predict the inflammatory profile seen in humans. Attempts have been made to identify reliable *in vivo* markers of reactogenicity in both animal models and humans, such as C-reactive protein (CRP),^20^ or a combination of CRP and IL6, respectively.^21^ However, this approach does not address the potential differences in the response to an adjuvanted vaccine by different species (reviewed in ref.^22^) or the translatability of findings to humans. Thus, methods are still needed that predict reactogenicity of novel adjuvants and adjuvant/antigen combinations *in vitro*. Ideally, identifying problematic novel adjuvant candidates at an earlier stage would be based on *in vitro* cell culture systems. Even when systems immunology approaches are applied to identifying *in vivo* profiles of reactogenicity,^23^ few attempts are made so far to correlate *in vivo* and *in vitro* reactogenicity profiles. While it is preferable to use a defined cell line (e.g., a human monocytic cell line),^24^ it remains to be shown whether a single cell type can reliably predict reactogenicity considering the differential expression of innate immune receptors on different cell types *in vivo*.

We are advocating for the establishment of a “reactogenicity score” that is based on a broader panel of *in vitro* markers (including not only pro- but also anti-inflammatory cytokines) to establish innate immune profiles that may predict reactogenicity. Since the antigen component of a vaccine can significantly contribute to a vaccine’s safety profile,^25^ it will be necessary to test adjuvants systematically, i.e., by themselves, with different antigens, and also the formulation that will be used *in vivo*. Licensed vaccines with different *in vivo* reactogenicity characteristics can be used to establish a “reactogenicity score” that would be linked to the vaccine’s *in vitro* reactogenicity profile. The reactogenicity scores from adjuvants used in licensed vaccines would be helpful in down-selecting novel adjuvants and adjuvant-antigen combinations. However, the reactogenicity score would still have several shortcomings, such as being limited to predicting reactogenicity after a primary immunization considering that adaptive recall responses can enhance innate immune stimulation and, thus, increase reactogenicity after a boost. The *in vitro* model would also not be able to address vaccine dose-related reactogenicity. As mentioned above, in the future, human organoid systems could also be a valuable tool for addressing adjuvant-induced reactogenicity that is enhanced due to pre-immune status.

## Adjuvant immunoprofiling: Species-specific responses

Animal studies are essential for determining the utility of vaccine formulations, but suffer from several shortcomings, most notably: 1) fine-specificities of PRRs differ between species;^26^ 2) PRR expression patterns differ across species (reviewed in ref.^27^) 3) responses to antigen and adjuvant are dose dependent, thus results obtained with an “optimized” vaccine dose in a small rodent model may not translate to a large animal model or humans; and 4) downstream signals induced by PRR agonists can be species-specific.^27,28^ Not surprisingly, the profiling of different adjuvants used for a SARS-CoV−2 antigen yielded significantly different immune profiles in mice and non-human primates,^29^ and using immune profiles obtained in immunized animals to predict immune profiles induced by the same vaccine in humans can be challenging. This observation complicates the characterization of vaccine-induced immune profiles when different host species are used and may call into question the value of down-selecting adjuvants for vaccine candidates in animal models. One approach to address species-specific responses to defined immunostimulators has been to create humanized mouse models, where the mouse PRR is replaced by its human counterpart, ideally not only using the PRR-gene but also flanking genomic sequences to include regulatory elements and obtain “human-like” gene expression (e.g., TLR4-Tg mice).^30^ Nevertheless, humanizing every component of the immune response to an adjuvanted vaccine is constrained by the complexity of the response and the number of regulatory pathways and circuits involved that cannot all be replaced.

To reconcile data obtained in different species, we are endorsing the concept of “humanizing computational models.”^31,32^ Mathematical and computational approaches that take into account species-specific differences in translational analyses would allow the identification of previously unappreciated networks and mechanisms that are critical for adjuvanticity and vaccine efficacy. Rather than aligning parallel pathways (such as the release of specific cytokines), this approach takes a holistic view of the overall immune profile the same vaccine induces in different species. This type of analysis can provide unprecedented insights into cellular interactions, tissue responses, pathways, adjuvant mechanisms of action (MoA), and also account for various modes of immunization to provide a better understanding of vaccine efficacy. In addition, it can potentially enable the dissecting of adjuvant properties in adjuvant comparison studies, and help “translate” findings in an animal model into corresponding human immune profiles *in silico*.

## Adjuvant immunoprofiling: Benchmark adjuvants and adjuvant comparison

Immunological profiles, or “fingerprints,” of adjuvants^33^ have limited value when generated for just individual adjuvants. They are most valuable when they can be compared to the profiles of other adjuvants, ideally obtained by using the same experimental protocols, and particularly when compared to “benchmark” adjuvants. Several studies have included alum as a comparator or benchmark adjuvant. However, no single adjuvant can serve as a universal benchmark due to the complexity of pathways and signals induced by different immunostimulators. Even the utility of alum as a benchmark adjuvant is limited by the fact that a variety of aluminum salt-based formulations are commercially available that do not have the same physiochemical characteristics and induce somewhat different immune profiles.^34^ Thus, in profiling studies, it may be advisable to use two distinct reference adjuvants.^34^ A reference adjuvant should meet at least two requirements: used in a licensed vaccine, and easy access to quality-controlled material. Including adjuvants used in licensed vaccines establishes “relevance” of the response measured and relying on a commercial source of the reference material assures that the same material is used across laboratories. Currently, these requirements limit the choice of reference adjuvants to aluminum salt-based adjuvants and Addavax, a commercially-available mimic of MF59.

The National Institute of Allergy & Infectious Diseases (NIAID) of the National Institutes of Health (NIH) continues to support research focused on the characterization of the immune response, the development of vaccines, and the discovery and development of novel adjuvants. To keep pace with the advances in vaccine design, to address the gap in knowledge regarding rational selection of adjuvants, and to leverage the power of computational modeling, NIAID launched two adjuvant comparison programs,^e^ Adjuvant Comparison & Characterization (ACC)^f^ and Advancing Vaccine Adjuvant Research for Tuberculosis (TB) (AVAR-T).^g^ At the core of the ACC and AVAR-T programs are side-by-side comparisons of vaccine adjuvants using the same vaccine and readout methods; bridging studies of adjuvant-induced immune profiles between host species; and the computational integration of immune profile data. These data will be made available to the scientific community through the NIAID ImmPort^h^ and Vaccine Adjuvant Compendium (VAC)^i^ to improve the pairing of vaccines and adjuvants.

## Notes

[a] Adjuvant identifier: VO_0005329 (https://vac.niaid.nih.gov/view?id=13)

[b] Adjuvant identifier for GLA-SE: VO_0005420 (https://vac.niaid.nih.gov/view?id=62)

[c] Adjuvant identifier: VO_0005207 (https://vac.niaid.nih.gov/view?id=37)

[d] Adjuvant identifier: VO_0005301 (https://vac.niaid.nih.gov/view?id=12)

[e]
https://www.niaid.nih.gov/research/adjuvant-comparison

[f]
https://www.niaid.nih.gov/research/adjuvant-comparison-characterization-acc

[g]
https://www.niaid.nih.gov/research/advancing-vaccine-adjuvant-research-tuberculosis-avar-t

[h]
https://www.immport.org/home

[i]
https://vac.niaid.nih.gov
